# Comparison between liver transplantation and resection for hilar cholangiocarcinoma: A systematic review and meta-analysis

**DOI:** 10.1371/journal.pone.0220527

**Published:** 2019-07-31

**Authors:** Dimitrios Moris, Ioannis D. Kostakis, Nikolaos Machairas, Anastasia Prodromidou, Diamantis I. Tsilimigras, Kadiyala V. Ravindra, Debra L. Sudan, Stuart J. Knechtle, Andrew S. Barbas

**Affiliations:** 1 Department of Surgery, Duke University Medical Center, Durham, NC, United States of America; 2 Department of Transplantation, Guy's Hospital, Guy's and St Thomas' NHS Foundation Trust, London, United Kingdom; 3 Third Department of Surgery, Attikon University Hospital, School of Medicine, National and Kapodistrian University of Athens, Athens, Greece; MD Anderson Cancer Center, UNITED STATES

## Abstract

**Background:**

Hilar cholangiocarcinoma (hCCA) is a rare and aggressive malignancy with R0 resection being currently the only option for long-term survival. With the improvement in the outcomes of liver transplantation (LT), the indications for LT have expanded to include other malignant tumors, such as hCCA. The aim of the present analysis is to demonstrate and critically evaluate the outcomes of LT compared to resection with curative intent in patients with hCCA.

**Methods:**

We systematically searched the literature for articles published up to May 2018. The following algorithm was applied ((hilar cholangiocarcinoma) OR (perihilar cholangiocarcinoma) OR klatskin$ OR (bile duct neoplasm) OR cholangiocarcinoma) AND (transplant$ OR graft$).

**Results:**

Neoadjuvant treatment with chemotherapy and radiation therapy was far more common in the LT group, with very few patients having received preoperative therapy in the resection group (p = 0.0005). Moreover, length of hospital stay was shorter after LT than after resection (p<0.00001). In contrast, no difference was found between the two treatment methods concerning postoperative mortality (p = 0.57). There was a trend towards longer overall survival after LT in comparison with resection. This was not obvious in the first year postoperatively, however, the advantage of LT over resection became obvious at 3 years after the operation (p = 0.02).

**Conclusions:**

In non-disseminated unresectable tumors, LT seems to have a non-inferior survival. In the same patients, neoadjuvant chemoradiotherapy and/or strict selection criteria may contribute to superior survival outcomes compared to curative-intent resection. Due to the scarcity of level 1 evidence, it remains unclear whether LT should be increasingly considered for technically resectable early stage hCCA.

## Introduction

Hilar cholangiocarcinoma (hCCA) is a rare and aggressive malignancy originating from the biliary epithelium at the confluence of the right and left hepatic ducts.[1] Treatment of the disease remains challenging and includes surgical resection combined with neoadjuvant or adjuvant therapies including external beam radiation therapy and systemic chemotherapy.[2, 3] Complete surgical resection (R0) is the only option for long-term survival, which varies from 20–40% at 5 years in most series.[4–6] Five-year survival rates above 50% has been reported in a subgroup of patients undergoing right trisectionectomy and portal vein resection.[7] Unfortunately, many individuals present with unresectable disease, owing to extensive hilar invasion, bilateral liver involvement, or vascular involvement. Additionally, many hCCAs arise from underlying primary sclerosing cholangitis (PSC), which limits the possibility of resection due to underlying parenchymal liver disease.[8]

During the early era of liver transplantation (LT) in the 1960s and 1970s, one of the primary indications for LT was for unresectable malignant liver tumors.[9] However, due to high recurrence rates and poor outcomes, LT for malignancy became much more limited thereafter, being reserved primarily for selected patients with early-stage primary hepatocellular carcinoma (HCC)[10]. Over the last few decades, as outcomes of LT have improved, the indications for LT have expanded to include other malignant conditions, including neuroendocrine liver metastases, colorectal liver metastases, and cholangiocarcinoma (both intrahepatic and hCCA).[11–15]

For patients with unresectable hCCA or hCCA arising in the setting of a chronic liver disease, LT is theoretically an attractive option, as it maximizes resection margin and cures the underlying parenchymal liver disease. Unfortunately, the early experience with LT alone for hCCA, which did not include any adjunctive therapy, was disappointing due to low survival rates.[16, 17]. The paradigm began to shift in the early 2000s when Sudan and colleagues at the University of Nebraska published a study demonstrating significant improvements in survival using a protocol combining neoadjuvant chemoradiation therapy with LT for patients with hCCA.[18] Building on this initial experience, the Mayo Clinic group established a neoadjuvant protocol including external beam radiation therapy together with intravenous fluorouracil (5-FU), followed by intraluminal brachytherapy and oral capecitabine. Additionally, prior to proceeding with LT, an exploratory laparotomy is performed to assess locoregional lymph nodes.[19] Of note, early reports with this approach conferred remarkably promising results, with a 5-year survival rate exceeding 80%.[19, 20] Yet, the more recent studies have noted a survival rate at the level of approximately 65–70%.[21]

While LT has evolved into an excellent therapy for selected patients with hCCA, it continues to be limited by the shortage of available donor organs. As such, surgical resection continues to be viewed as first-line therapy if technically feasible. The aim of the present analysis is to demonstrate and critically evaluate the outcomes of LT compared to resection with curative intent in patients with hCCA.

## Methods

### Search strategy and eligibility of the studies

This systematic review and meta-analysis followed the Preferred Reporting Items for Systematic Reviews and Meta-Analysis (PRISMA) guidelines (S1 Table) and was conducted based on the authors’ predetermined inclusion criteria.[22] Of note, three authors (DM, IDK and NM) screened the literature independently. All prospective and retrospective studies published in English, which reported outcomes of patients who underwent LT versus resection for hCC were considered eligible for inclusion in the present meta-analysis. Reviews, preclinical studies and case reports were excluded from the present meta-analysis. Additionally, original articles referring only to LT, resection or other treatment modalities as well as non-English studies were excluded. Discrepancies during the data collection were resolved by consensus among all authors.

### Literature search and data collection

The following databases were searched for articles published from January 2000 up to May 2018: Ovid Medline, Embase, Scopus, Google Scholar, Clinical Trials and Cochrane Central Register of Controlled Trials. Reference lists of articles, which were retrieved in full text, were additionally systematically searched for relevant articles in the field. The search terms used included: “hilar’, “perihilar”, “bile duct neoplasm”, “cholangiocarcinoma”, “Klatskin”, “grafting$”, “transplant$”, “liver transplantation”, “resection” and “hepatectomy”, which created the following algorithm: ((hilar cholangiocarcinoma) OR (perihilar cholangiocarcinoma) OR klatskin$ OR (bile duct neoplasm) OR cholangiocarcinoma) AND (transplant$ OR graft$). Our search strategy included the MeSH terms shown in S2 Table.

### Variables

We extracted data about patient number, gender, age, neoadjuvant treatment, tumor size, stage and grade, Bismuth-Corlette classification, the presence of involved lymph nodes, resection margin status, duration of operation, blood loss, length of hospital stay, postoperative morbidity, postoperative mortality, disease-free survival and overall survival.

### Quality Assessment

The Methodological Index for Non-Randomized Studies (MINORS) was used to assess the quality of the included studies.[23] MINORS is a quality assessment tool, designed for estimating the methodological adequacy of non-randomized studies and contains 12 items, each scored from 0 to 2, thus providing overall scores ranging from 0 to 24. The use of the MINORS scale was used due to the fact that all of the studies included in our meta-analysis were non-randomized.

### Statistical analysis

Risk ratio (RR), mean differences and hazard ratio (HR) were used to examine categorical, continuous and survival, respectively. Due to the expected heterogeneity among the included studies, the random effects model was chosen. Comparisons between dichotomous or continuous variables were made with the inverse variance method, while comparisons regarding survival were made with the generic inverse variance method. Statistical heterogeneity was assessed with the Higgin’s I^2^ statistic. 95% confidence intervals (CI) were noted for all results. Mean values and standard deviations were calculated according to the equations proposed by Hozo et al.[24] when they were not mentioned in the studies. Furthermore, log HR and its standard error (SE) were calculated according to the equations proposed by Parmar et al.[25] when they were not reported in the studies. The level of statistical significance was set at a = 0.05. All analyses were performed using the Review Manager Version 5.3 software.

## Results

### Search outcomes

**Fig 1:**
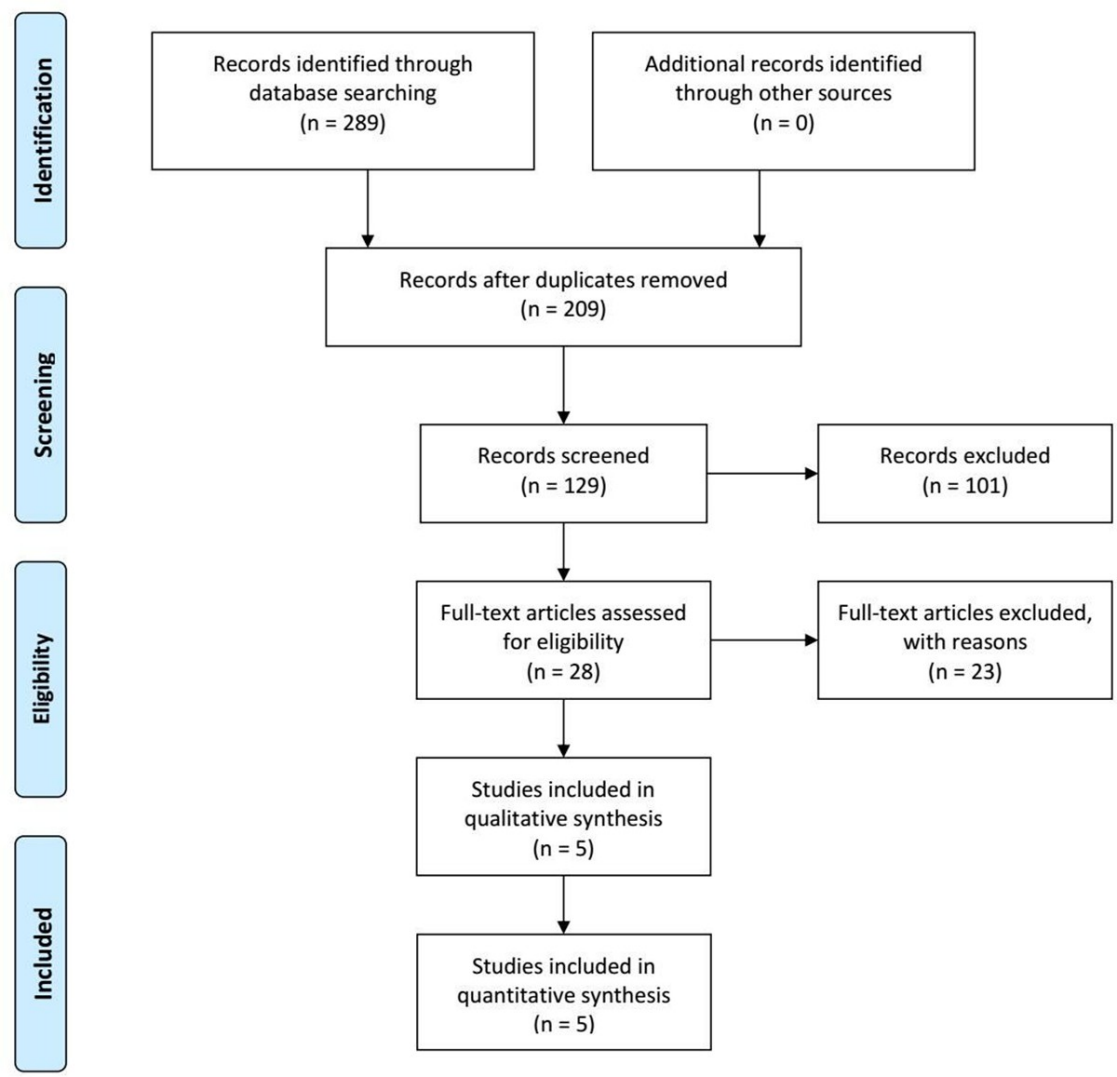
The search strategy of the present analysis.

Out of the 2592 records screened during the database search, 289 were retrieved in full text, among which 281 did not meet the inclusion/exclusion criteria (Fig 1). Of the remaining 8 studies [26–33], 5 reports were considered eligible for inclusion, whereas the remaining 3 were excluded with reasons; one was excluded due to overlapping patient population [31], one was published prior to 2000 [33] and one due to lack of separate reported data for patients undergoing LT for hCC [32]. Two studies were conducted in the USA [28, 29], one in Germany [27], one in the United Kingdom [26] and one in Spain.[30] All the included studies were retrospective.[26–30] As far as the quality assessment of the included studies is concerned, the MINORS scale provided moderate scores ranging between 14 and 17, as can be seen in Table 1.

**Table 1:** Study characteristics.

Study	Country	MINORS	Transplantation (patients)	Resection (patients)	Indications for the transplantation arm	Neoadjuvant treatment before transplantation	Staging operation	Median follow-up
2018; Ethun[29]	USA	17	41	191	Locally unresectable hilar cholangiocarcinoma (PSC and non-PSC patients)	Yes (39/41)	Yes	Entire cohort: 23 months, transplantation: 58 months, resection: 15 months
2015; Croome[28]	USA	17	54	99	Locally unresectable hilar cholangiocarcinoma (PSC and non-PSC patients)	Yes (54/54)	Yes	Transplantation: 43 months, resection: 26 months
2010; Kaiser[27]	Germany	15	7	7	Hilar cholangiocarcinoma	No	No	Entire cohort: 32 months
2008; Hidalgo[26]	UK	17	12	44	Locally unresectable hilar or perihilar cholangiocarcinoma (PSC and non-PSC patients)	No	Yes (since 2000) (study period: 1993–2003)	Entire cohort: 21.7 months
2007; Robles[30]	Spain	14	10	23	Locally unresectable hilar cholangiocarcinoma	No	Yes	N/A

MINORS: Methodological index for non-randomized studies, N/A: not available

### Patient characteristics

There were 488 patients with hilar cholangiocarcinoma in total in the 5 included studies; 124 underwent liver transplantation and 364 underwent resection.[26–30] There was a higher rate of male patients in the group of liver transplantation in comparison with the resection group [transplantation: 81/114 (71.1%), resection: 203/341 (59.5%), RR: 1.18, 95% CI: 1.02 to 1.37, p = 0.03; I^2^: 0%, p = 0.98].[26–29] Furthermore, the patients who underwent liver transplantation were younger on average than the patients who underwent resection (mean difference: -8.92 years, 95% CI: -12.94 to -4.9, p<0.0001).[26–29] However, there was significant heterogeneity among studies regarding patient age (I^2^: 75%, p = 0.008).[26–29] In addition, neoadjuvant treatment with chemotherapy and radiation therapy was far more common in the transplantation group, with very few patients having received preoperative therapy in the resection group [transplantation: 93/102 (91.2%), resection: 9/297 (3%), RR: 41.88, 95%CI: 5.19 to 337.8, p = 0.0005; I^2^: 60%, p = 0.11].[27–29]

### Tumor characteristics

There were no significant differences between the two treatment methods as far as tumor characteristics are concerned.[26–30] First, there was no significant difference in terms of tumor size between the transplantation and the resection group (mean difference: -0.51 cm, 95% CI: -1.79 to 0.77, p = 0.44), although there was significant inter-study heterogeneity (I^2^: 93%, p<0.00001).[26, 28, 29] Moreover, there was similar distribution of advanced stages (T3 or T4 tumors) between the two groups [transplantation: 20/46 (43.5%), resection: 67/198 (33.8%), RR: 0.99, 95% CI: 0.73 to 1.34, p = 0.95; I^2^: 0%, p = 0.8].[26, 27, 29] However, there was a higher rate of tumors categorized as Bismuth-Corlette IV in the transplantation group than in the resection group [transplantation: 64/84 (76.2%), resection: 82/282 (29.1%)], even though this difference did not reach statistical significance (RR: 1.46, 95% CI: 0.73 to 2.94, p = 0.28; I^2^: 78%, p = 0.01).[27–29] Similarly, the differences noticed regarding the rates of involved lymph nodes between the two groups were comparable [transplantation: 15/124 (12.1%), resection: 127/364 (34.9%), RR: 0.43, 95% CI: 0.14 to 1.36, p = 0.15], but again with great heterogeneity among studies (I^2^: 81%, p = 0.006).[26–30]. In addition, there was no significant difference in terms of the presence of high grade tumors between the two treatment methods [transplantation: 33/90 (36.7%), resection: 122/319 (38.2%), RR: 0.93, 95% CI: 0.34 to 2.6, p = 0.9], even though there was high inter-study heterogeneity (I^2^: 87%, p = 0.0004).[26, 28, 29]

### Operative and postoperative outcomes

**Fig 2:**
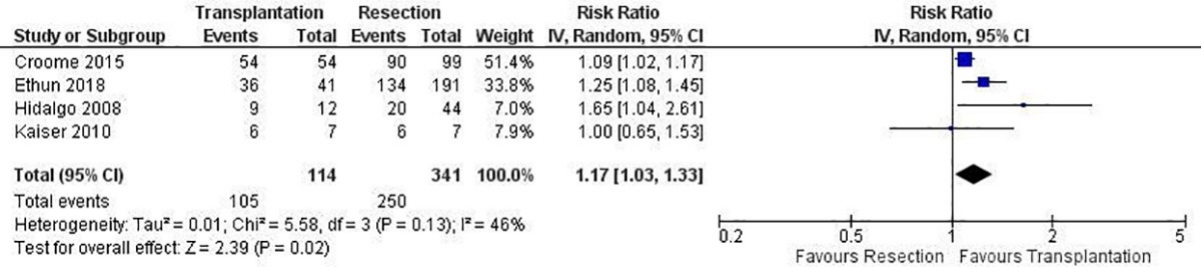
The comparison of R0 resection rates between resection and transplantation.

There was a higher rate of R0 resections in the transplantation group than in the resection group [transplantation: 105/114 (92.1%), resection: 250/341 (73.3%), RR: 1.17, 95% CI: 1.03 to 1.33, p = 0.02; I^2^: 46%, p = 0.13][26–29] (Fig 2). Moreover, length of hospital stay was shorter after transplantation than after resection (mean difference: -2.8 days, 95% CI: -3.46 to -2.13, p<0.00001; I^2^: 0%, p = 0.5).[27, 29] In contrast, there was no difference between the two treatment methods concerning postoperative mortality [transplantation: 3/48 (6.3%), resection: 16/198 (8.1%), RR: 0.7, 95% CI: 0.2 to 2.44, p = 0.57; I^2^: 0%, p = 0.75].[27, 29] Only the study conducted by Ethun et al. provided information about postoperative morbidity. In particular, transplantation resulted in lower rates of postoperative complications overall when compared with resection (transplantation: 49%, resection: 68%, p = 0.03), but this difference was not significant when major complications were considered (transplantation: 34%, resection: 45%, p = 0.3).[29]

### Survival

**Fig 3:**
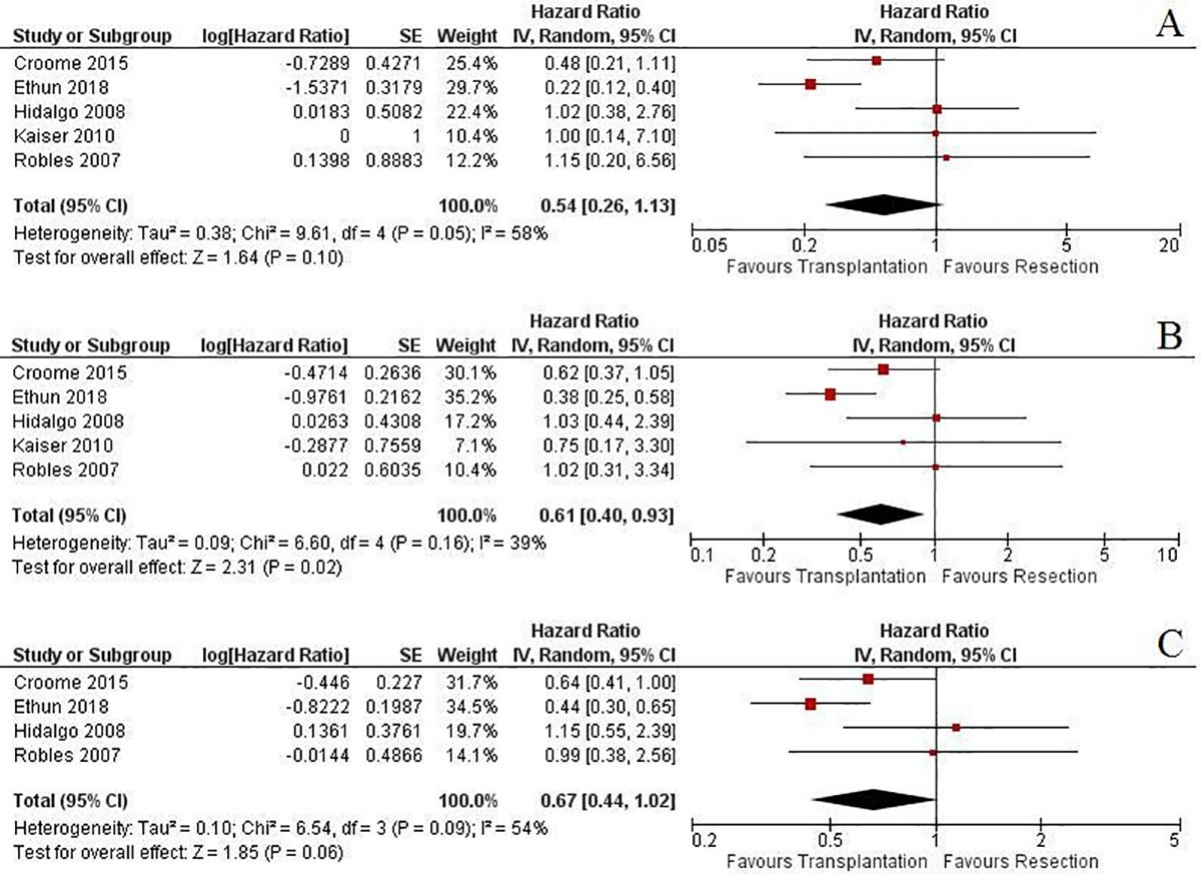
The comparison of A) 1-year, B) 3-year and C) 5-year overall survival rates between resection and transplantation.

There was a trend towards longer overall survival after transplantation in comparison with resection.[26–30] This was not obvious in the first year postoperatively (HR: 0.54, 95% CI: 0.26 to 1.13, p = 0.1; I^2^: 58%, p = 0.05)[26–30] (Fig 3A). However, the advantage of transplantation over resection became obvious at 3 years after the operation (HR: 0.61, 95% CI: 0.4 to 0.93, p = 0.02; I^2^: 39%, p = 0.16)[26–30] (Fig 3B), and remained at 5 years after the operation, but did not reach statistical significance (HR: 0.67, 95% CI: 0.44 to 1.02, p = 0.06; I^2^: 54%, p = 0.09)[26, 28–30] (Fig 3C).

Two of the studies also performed an intention-to-treat analysis. In particular, Croome et al. reported a longer overall survival in patients who underwent LT compared with patients who underwent resection (p = 0.007) [28]. Similarly, Ethun et al. reported a median OS of 77.4 months for the patients listed for LT versus 27.4 months for the patients with attempted resection (p = 0.002).[29] When we excluded the two studies with the rigorous Mayo transplant protocol involving neoadjuvant therapy and a diagnostic laparoscopy,[27, 29] no significant difference regarding overall survival was detected at 1 (HR: 1.04, 95% CI: 0.47 to 2.3, p = 0.92; I^2^: 0%, p = 0.99), 3 (HR: 0.97, 95% CI: 0.52 to 1.81, p = 0.92; I^2^: 0%, p = 0.93) and 5 (HR: 1.08, 95% CI: 0.6 to 1.94, p = 0.79; I^2^: 0%, p = 0.81) years postoperatively.[26, 27, 30] This probably reflects the beneficial effect of neoadjuvant treatment on survival. Moreover, three studies noted that they included both patients with PSC and patients without PSC.^26,28,29^ Nevertheless, only the study conducted by Ethun et al. provided separate results for the non-PSC cases and found that the longer overall survival for the LT arm remained (median survival not reached yet vs 25.9 months; 95% CI 13.0–38.7, p = 0.03).^29^ Finally, only the study conducted by Croome et al. provided actuarial rates of progression-free survival. In particular, progression-free survival was longer after transplantation than after resection (p<0.001), with higher progression-free survival rates at 1, 3 and 5 years postoperatively (87%, 64%, 54%, respectively, in the transplantation group versus 74%, 48%, 29%, respectively, in the resection group).[28]

## Discussion

hCCA is classified anatomically according the modified Bismuth-Corlette classification. In brief, type I tumors involve the common hepatic duct distal to the biliary confluence and type II involve the biliary confluence. Type IIIa tumors involve the biliary confluence and the right hepatic duct, type IIIb tumors involve the biliary confluence and the left hepatic duct, whereas type IV tumors extend to the bifurcation of both the right and the left hepatic duct.[1] Type IV hCCA was traditionally considered unresectable due to the involvement of both hepatic ducts. However, there are published series in which resection of type IV hCCA was feasible, but technically demanding and with high morbidity. Nevertheless, these resections led to improved survival rates in case of N0M0 disease.[34] At this point, we should underline that the most important determinant of hCCA survival is the ability to perform an R0 resection.[1, 3, 35, 36]. Unfortunately, 30–40% of patients with hCCA are inoperable at diagnosis. The remaining patients may be eligible for surgery; yet approximately 30% of them are ultimately deemed non-resectable and have a prognosis similar to that of inoperable patients.[37] In the current era, these non-resectable cases can be referred for consideration of LT, provided that liver metastases, lymph node dissemination, or extrahepatic spread are not present.[1, 21]

The initially poor outcomes of LT for hCCA (30% 5-year survival) were attributed to specific factors such as poor patient selection, inadequate preoperative staging, and lack of neoadjuvant therapy.[18, 35] The subsequent development of neoadjuvant chemoradiation protocols and strict patient selection criteria by the Nebraska and Mayo Clinic groups has more recently produced a significant improvement in outcomes of LT for hCCA.

In theory, LT exhibits significant advantages when compared with conventional resection; indeed, LT has the potential to achieve complete resection of the tumor, by removing all hilar neural and lymphatic tissue. In addition, LT also does not require preservation of arterial and portal venous inflow to the remaining liver during surgery. The most serious disadvantages of the liver transplant hCCA treatment protocol at present are the limitation of donor organ availability and the need for long term immunosuppression and its attendant side effects.[38] The current therapeutic models combining principles of surgical oncology with transplantation exemplify the hybrid paradigm of “Transplant Oncology” aiming to treat or even cure complex diseases in a multidisciplinary framework. While these novel approaches appear promising, they require rigorous clinical testing to ensure proper patient selection and an ethical distribution of limited organs.[39]

In the United States, studies in which patients had LT after neoadjuvant chemoradiation have demonstrated marked improvements in survival.[18, 19, 40] Survival rates of 65% after 5 years and 59% after 10 years with recurrence rates of 20% have been shown after neoadjuvant treatment.[19, 40, 41] The same trend has been noted in the treatment of intrahepatic cholangiocarcinoma (iCCA) as well. A recent prospective study demonstrated that in patients with anatomically unresectable iCCA who had received neoadjuvant chemotherapy while awaiting LT, overall survival was 100% at 1 year, 83.3% at 3 years, and 83.3% at 5 years after LT, with 50% recurrence-free survival at 1, 3, and 5 years.[42] These findings were in line with another study on hCCA, where survival was higher after LT compared with curative-intent resection (P = 0.022).[31] Of interest, LT was correlated with a lower incidence of tumor recurrence than for patients undergoing resection (13% versus 27%).[31] Importantly, it has to be noted that the minimal survival benefit/expectation related to liver transplantation is a 50% survival at 5 years,[43] which was achieved in the aforementioned studies, as well as in the two larger studies included in our analysis (63.4% in the study by Ethun et al.[29] and 59.3% in the study by Croome et al.[28]).

The present analysis has some interesting findings. We identified a tendency towards longer overall survival after transplantation in comparison with resection at all time points, which reached statistical significance at 3 years after the operation. In addition, progression-free survival seems to be significantly longer after transplantation than after resection. Current results of LT for patients with hCCA seem to be comparable to that for patients with other primary and metastatic liver malignancies. We recently reported the results of an analysis on the role of LT to treat neuroendocrine tumors. We showed that recurrence after LT ranged from 31.3% to 56.8% and the reported 1-, 3-, and 5-year overall survival was 89%, 69%, and 63%, respectively.[14] Similarly, in patients with unresectable colorectal liver metastases, our analysis showed that the survival rates LT can reach 85.2%, 48% and 36.5% at 1-, 3-and 5 years respectively.[13] Finally, for hepatocellular carcinoma (HCC), patients within the Milan criteria reached a 1-year OS of 84.4% and a 5-year of 59.3% respectively. When compared to resection, patients with HCC after LT demonstrated longer 3-year disease-free survival (54.4% vs 74.2%, P  =  0.02).[44] However, as far as this meta-analysis is concerned, we cannot overstress the fact that the patients in the transplantation arm had locally unresectable disease, whereas the patients in the resection arm had resectable disease. Furthermore, it should be noted that although the difference in involved lymph nodes was not statistically difference, there were numerically more involved lymph nodes in the resection than in the transplantation group (34.9% vs 12.1%). This higher rate of positive lymph nodes may be at least partially responsible for the shorter survival in the resection group, since the presence of infiltrated lymph nodes has long been considered an independent predictor of disease recurrence and worse survival in patients with cholangiocarcinoma.[45] In contrast, there was no significant difference between the two treatment arms regarding the presence of high-grade tumors, which have been associated with worse survival rates.[26, 28] For all these reasons, the aforementioned results should be interpreted with caution.

One of the striking findings of our analysis was that the difference in patients treated with neoadjuvant therapy was dramatic between the two groups. The neoadjuvant treatment regimens that were applied in the two largest of the included studies were based on the Mayo protocol, namely the studies conducted by Croome et al.[28] and Ethun et al.[29]. More specifically, the treatment started with administration of external beam radiation (4500 cGy in 30 fractions) and concomitant intravenous administration of 5-FU (500 mg/m^2^ as a daily bolus) for the first 3 days of radiation. Two to three weeks following the completion of the external radiotherapy, additional transluminal radiation was given using a transcatheter Iridium-192 brachytherapy wire (2000–3000 cGy), although this step was omitted in several cases in the study by Ehun et al. After brachytherapy, patients continued chemotherapy with either intravenous 5-FU (500 mg/m^2^ per day) via an infusion pump or oral capecitabine (2000 mg/m^2^ per day in two divided doses, two out of every three weeks) until transplantation.[28, 29] Neoadjuvant chemoradiation has not been widely adopted in patients who are undergoing surgical resection due to the known adverse effects of radiation on hepatic parenchyma and the hilar structures which could impair the safety of dissection and reconstruction.[46, 47] When we performed a subgroup analysis of the pooled patients without neoadjuvant treatment, the survival benefit of LT was lost. Neoadjuvant chemoradiation appears to be an important element in improving survival outcomes after LT, but it is hard to determine whether the improved survival outcomes observed in the Mayo protocol can be solely attributed to the neoadjuvant therapy or the strict patient selection criteria (i.e. node negative patients with stable disease over time). Mantel et al. demonstrated that patients selected by the same criteria used in the Mayo protocol who underwent LT without the use of neoadjuvant chemoradiation also have favorable survival outcomes. Their analysis showed significantly increased 5-year survival rates of 59% in the Mayo Clinic criteria compliant group (p = 0.001), which closely resemble reported survival outcomes by Mayo Clinic for patients with pretreatment pathological confirmation of hCCA.[48] Unfortunately their study was limited by the lack of direct comparison to a group also fulfilling the Mayo Clinic criteria that had received neo-adjuvant treatment. Regardless, the study implied that stricter selection of patients alone might positively impact survival outcomes. The importance of selection criteria can also be deduced by the similar overall survival rates found by Croome et al. in patients with N0, R0 disease undergoing either LT or resection,[28] which however was not confirmed by Ethun et al, who showed longer overall survival after LT even in this subgroup of patients.[29]

The included studies in our analysis were non-randomized and retrospective. However, there is an ongoing French study, the TRANSPHIL study (NCT02232932) [49], which is a prospective, randomized, multicenter one for hilar cholangiocarcinoma, comparing an interventional group with liver transplantation and neoadjuvant radio-chemotherapy and a control group with conventional liver and bile duct resection. The inclusion criteria are similar to those defined by the Mayo group, apart from the fact that the patients undergoing liver transplantation need to have potentially resectable disease and that these with PSC are excluded. In addition, no intraluminal brachytherapy will be performed and the external radiotherapy is slightly higher than the Mayo regimen, at 50 Gy. This study will provide a much more definitive answer comparing the two modalities as it will be comparing LT and resection in the same group of patients, whereas the current meta-analysis involves unresectable patients in the LT arm. The TRANSPHIL study will also require patients to undergo appropriate extensive staging evaluation, including PET and staging laparotomy.

Our study presents some inherent limitations that need to be addressed. First, all studies included were non-randomized, retrospective analyses and thus subject to the attendant biases. In particular, there was heterogeneity in terms of neoadjuvant treatment and whether tumors were associated with PSC or arose de novo. More specifically, only one study had available data for patients with and without PSC.[29] The other studies did not provide sufficient granularity on this aspect. It should be noted that including PSC patients in this analysis may skew results towards longer overall survival with LT, since they are generally diagnosed at an earlier stage resulting in improved outcomes. Furthermore, the patients in the transplantation arm had locally unresectable disease, while the patients in the resection arm had resectable disease. In addition, there was heterogeneity among studies concerning staging and evaluating the extent of the disease. To be more specific, only Croome et al.[28] and Hidalgo et al.[26] reported that they used magnetic resonance imaging (MRI) as part of preoperative staging and Croome et al.[28] also mentioned that they used endoscopy to identify the extent of the tumor in the distal bile duct. Moreover, we should note that all median follow-up times were below 5 years (Table 1) and not many patients were follow-up up to 5 years postoperatively or more. Therefore, conclusions about 5-year survival rates should be considered with caution. Finally, the small number of studies, and therefore patients, included in the analysis highlight the necessity for follow-up comparative studies.

## Conclusions

In non-disseminated unresectable tumors, liver transplantation seems to have a non-inferior survival. In these same patients, neoadjuvant chemoradiotherapy and strict selection criteria may contribute to superior survival outcome compared to curative-intent resection. Due to the scarcity of available organs and the lack of level 1 evidence, it remains unclear whether LT should be increasingly considered for technically resectable early stage hCCA.

## Supporting information

S1 Table Prisma Checklist. (DOCX)

S2 Table MeSH terms used in the search strategy. (DOCX)

S1 Data Data from individual studies analyzed. (XLSX)
