# Supplementary material for: Comparison between liver transplantation and resection for hilar cholangiocarcinoma: A systematic review and meta-analysis
Source: PLoS One. 2019 Jul 31;14(7):e0220527. doi: 10.1371/journal.pone.0220527 (PMC6668826; doi:10.1371/journal.pone.0220527)
Supplement: S2 Table — (DOCX) [file pone.0220527.s002.docx]

| - (("klatskin tumor"[MeSH Terms] OR ("klatskin"[All Fields] AND "tumor"[All Fields]) OR "klatskin tumor"[All Fields] OR ("hilar"[All Fields] AND "cholangiocarcinoma"[All Fields]) OR "hilar cholangiocarcinoma"[All Fields]) OR ("klatskin tumor"[MeSH Terms] OR ("klatskin"[All Fields] AND "tumor"[All Fields]) OR "klatskin tumor"[All Fields] OR ("perihilar"[All Fields] AND "cholangiocarcinoma"[All Fields]) OR "perihilar cholangiocarcinoma"[All Fields]) OR klatskin$[All Fields] OR ("bile duct neoplasms"[MeSH Terms] OR ("bile"[All Fields] AND "duct"[All Fields] AND "neoplasms"[All Fields]) OR "bile duct neoplasms"[All Fields] OR ("bile"[All Fields] AND "duct"[All Fields] AND "neoplasm"[All Fields]) OR "bile duct neoplasm"[All Fields]) OR ("cholangiocarcinoma"[MeSH Terms] OR "cholangiocarcinoma"[All Fields])) AND (("transplants"[MeSH Terms] OR "transplants"[All Fields] OR "transplant"[All Fields] OR "transplantation"[MeSH Terms] OR "transplantation"[All Fields]) OR ("transplants"[MeSH Terms] OR "transplants"[All Fields] OR "graft"[All Fields])) |
| --- |

**S2 Table**. MeSH terms used in the search strategy
